# TWO STEPS FORWARD AND THREE STEPS BACK: STUDENTS’ PERSPECTIVES ON LIVING AND WORKING WITHIN AN AGING SOCIETY

**DOI:** 10.1093/geroni/igz038.2461

**Published:** 2019-11-08

**Authors:** Catherine J Tompkins, Emily S Ihara, Katelyn Bittinger

**Affiliations:** George Mason University, Fairfax, Virginia, United States

## Abstract

Each day, 10,000 Americans celebrate a 65th birthday, but there are still many young adults not choosing to enter a field that focuses on working with the older population. As part of a continued effort to understand low enrollments in a minor in aging studies and a graduate certificate program in gerontology, focus groups were held with students to explore why they are not choosing to learn more about one of the fastest growing sectors of the U.S. population. A total of 21 students participated in two focus groups. Students’ majors varied but included social work, public health, nursing and communications. All of the students were between the ages of 18 and 22 except for one student who was 68 years old. Only 6 students had taken an aging class, but every student indicated that they were close to a family member, 60 years old or older. Two researchers coded and analyzed the focus group data for themes. Examples of the themes about older adults included being unwilling to change, having negative views toward millennials, and being hampered by technology. Themes relative to student perspectives included needing empathy and patience to work with older adults, assuming decline and lacking opportunities within their majors to take gerontology courses. Comparing views and perspectives of older adults decades ago by traditional college-aged students to current day perspectives resulting from this data will be discussed as well as successful strategies for increasing enrollments in gerontology programs.

